# Problem-based or lecture-based learning, old topic in the new field: a meta-analysis on the effects of PBL teaching method in Chinese standardized residency training

**DOI:** 10.1186/s12909-022-03254-5

**Published:** 2022-03-31

**Authors:** Tingting Li, Weidong Wang, Zhijie Li, Hongmiao Wang, Xiaodan Liu

**Affiliations:** Department of Nephrology, The First Affiliated Hospital of China Medical University, 155 North Nanjing Street, Shenyang, Liaoning 110001 P.R. China

**Keywords:** Standardized residency training, problem-based learning, lecture-based learning, meta-analysis

## Abstract

**Background:**

Standardized residency training (SRT) is crucial for graduate medical education and the training of high-quality doctors. Nevertheless, China started SRT nationwide only in the recent decade. During these years, researchers have been searching for suitable teaching methods to improve the abilities of residents. Although the problem-based learning (PBL) teaching mode has been applied in undergraduate teaching for many years, the teaching effect of PBL has not been unified in Chinese SRT according to the core competences of the residents.

**Methods:**

Studies that compared the teaching effect of PBL and lecture-based learning (LBL) on SRT in China from January 2010 to April 2020 in the Chinese databases, such as China National Knowledge Infrastructure (CNKI), WanFang, WeiPu, Chinese BioMedical Literature (CBM), and English-language online databases, such as PubMed, Embase, and Cochrane Library were systematically reviewed. Data were analyzed using the Stata version 12.0 software.

**Results:**

A total of 75 articles (76 studies) were included in this meta-analysis. Compared with LBL group, PBL-based methods are more effective in the mastery of medical theory knowledge (WMD = 7.14, 95% CI: 5.93–8.34), operational skills (WMD = 6.54, 95% CI: 4.55–8.53), analysis and diagnosis of cases (WMD = 8.52, 95% CI: 7.50–9.53), and overall capacity (WMD = 8.70, 95% CI: 6.87–10.53), but showed no advantage on operational skills in diagnostic imaging (WMD = 1.30, 95% CI: -0.11–2.71). The questionnaire surveys analyzed in this meta-analysis indicated the positive effects of PBL on the mastery of theoretical knowledge, clinical diagnostic thinking, teamwork ability, ability to analyze and solve problems, ability to consult documents, learning interest and learning efficiency, but that there were no advantages in improving self-directed learning ability, communication ability and hands-on ability. The questionnaire result analyzed in this meta-analysis also showed the residents’ satisfaction with PBL-based strategies.

**Conclusions:**

Taken together, the current meta-analysis provides a systematic and comprehensive analysis on PBL teaching mode in Chinese SRT and outlines a path for further research on the detailed design of suitable teaching methods for different specialties and abilities.

**Supplementary Information:**

The online version contains supplementary material available at 10.1186/s12909-022-03254-5.

## Introduction

Standardized residency training (SRT) occupies a very important role in connecting the basic education of medical colleges and continuing medical education, and it is the key tool and pathway to train qualified clinicians for improving the overall medical level. However, the unified nationwide reform of medical education about SRT in mainland China did not begin until 2013, termed as 5 + 3 model, encompassing 5 years of undergraduate medical studies (leading to a Bachelor degree) and 3 years of SRT in one of the 36 specialties [1, 2]. Compared to the developed countries, wherein the SRT has been gradually maturing after a hundred years, this program is still in its infancy [3, 4].

With increasing focus on cultivation of competencies which is the critical problem of SRT, selecting a suitable type of teaching method is needed urgently [5, 6]. Problem-based learning (PBL), of which the training objectives are consistent with those of resident trainees, has been carried out in some residency training bases in recent years [7, 8]. However, whether PBL is better than lecture-based learning (LBL) which is the primary teaching method in the Chinese medical education system, there still is no uniform conclusion [9].

A common limitation of previous studies on this topic is that they all include the research before the nationwide reform of SRT [10, 11]. Before performing SRT, majority of the medical students have been directly engaged in clinical work in hospitals at different levels, and it has a severe impact on the homogenization training of the residents without unified standards. The SRT with guidelines for each specialty rotation about required time, purpose, requirements and assessments, would ensure that medical school graduates receive standardized and institutionalized training in the certified training institutions [12]. Therefore, it is of great importance to analyze the effects of PBL vs. LBL teaching method under the unified background of SRT reform.

Herein, we present a meta-analysis for the first time which only includes the studies performed under the nationwide reform of SRT. In addition, this meta-analysis includes the mastery of medical theory knowledge, operational skills, and analysis and diagnosis of the cases, while subgroup analyses based on the teaching methods and department types were also carried out. Moreover, questionnaire surveys (QS) about theoretical knowledge mastery, clinical diagnostic thinking, teamwork ability, ability to analyze and solve problems, communication ability, learning interest, self-directed learning ability, hands-on ability, ability to consult documents, learning efficiency, and satisfaction with teaching were systematically analyzed. Together, this accurate and comprehensive analysis would provide a scientific basis for the selection and application of teaching methods in Chinese SRT in the future.

## Methods

### Literature search

We searched China National Knowledge Infrastructure (CNKI), WanFang (Chinese database), WeiPu (Chinese database), Chinese BioMedical Literature (CBM), and English-language online databases, such as PubMed, Embase, and Cochrane Library. The following terms or keywords were used: “problem-based learning” OR “PBL”) AND (“case-based learning” OR “CBL”) AND (“standardized residency training” OR “standardized training” OR “SRT” OR “resident” OR “5 + 3 model”. Next, the references of the review articles were scanned for additional eligible reports. The search was restricted from January 2010 to April 2020; no language restrictions were imposed.

### Inclusion criteria

The studies were included according to the following four criteria: (a) Target population: residents in SRT in China; (b) Study design: randomized controlled trials; (c) Interventions: PBL or PBL + CBL served as the experimental group and LBL comprised the control group; (d) Outcome measurements (at least one of these): knowledge scores (KS), were used to assess how well the residents mastered the related theoretical knowledge; skill scores (SS), which were used to assess the operational skills, such as urethral catheterization in Urology and endotracheal intubation in Anesthesiology; practical skills (PS) assessments, including medical history collection, physical examination, making diagnosis and treatment plan, were used to assess the ability of solving practical clinical problems; total scores (TS), which included knowledge scores, skill scores and practical skills scores, were used to assess the overall abilities; QS, which were self-reported questionnaire surveys, were used to assess the residents' recognition of the relevant aspects of the different teaching methods. The results of SS, PS and TS were presented as scores out of 100 and for QS, in which each item was assessed by a yes or no, the results were presented as percentages.

### Exclusion criteria

The exclusion criteria were as follows: (a) Comprising of subjects other than residents; (b) The studies were non-randomized and non-controlled; (c) Utilized interventions other than PBL or PBL + CBL; (d) The control group was not LBL or combined with other methods; (e) No comparison of baseline indicators between the two groups; (f) Studies with partial data duplication.

### Data extraction

Data were independently extracted by two reviewers. Any disagreements about the eligibility were resolved by consensus. The following information was extracted for each included study: (a) the first author, (b) the year of publication, (c) the study type, (d) the sample size (intervention and control groups), (e) the specialty of the residents, (f) characteristics of the residents, (g) characteristics of the tutors, (h) the intervention methods, (i) year of residency training, (j) the duration of intervention, and (k) the outcome measures.

### Quality assessment

The quality of each included study was assessed using the risk of bias table according to the Cochrane Collaboration by two reviewers independently [13]. Any disagreement was resolved by discussion to achieve a consensus. The following quality items were checked: (a) random sequence generation, (b) allocation concealment, (c) blinding of participants and personnel, (d) blinding of outcome assessment, (e) incomplete outcome data, (f) selective reporting, and (g) other sources of bias.

### Statistical analysis

Data were analyzed using the Stata version 12.0 software. The effect sizes on scores were presented by weighted mean difference (WMD) and 95% confidence intervals (CIs), and those on questionnaires were presented by odds ratios (ORs) and 95% CIs. The chi-squared test-based Q-statistic and I^2^ statistic was used to estimate the heterogeneity (I^2^ ≤ 25%, low heterogeneity; 25% < I^2^ < 50%, moderate heterogeneity; I^2^ ≥ 50%, and high heterogeneity) [14]. A fixed-effects model was used to pool the results when heterogeneity was ≤ 50%, while a random-effects model was applied when heterogeneity was > 50% [15, 16]. Sensitive analysis was performed to investigate the influence of a single study on the overall pooled estimate by sequential deletion of each study. Subgroup analysis according to teaching methods and departments was conducted. The publication bias was evaluated by the Begg’s and Egger’s test [17, 18]. *P* < 0.05 indicated statistically significant publication bias.

## Results

### Search results

**Fig. 1:**
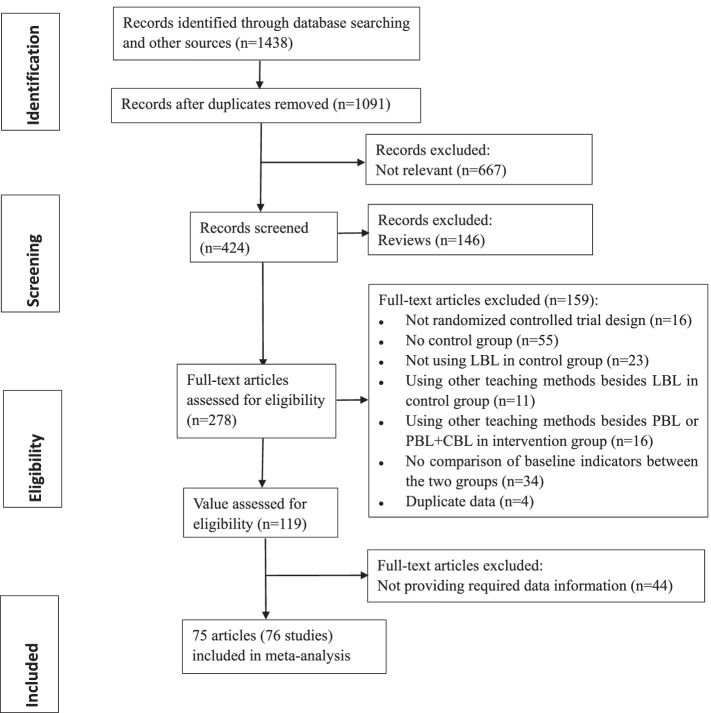
Schematic of the search strategy

The flow diagram of the search strategy is illustrated in Fig. 1. A total of 1438 potentially relevant articles was identified, of which 347 duplicates were removed. At the screening stage, 813 articles were excluded after reading the titles and abstracts, among which 667 were not relevant to the topic, and 146 were reviews. According to the inclusion and exclusion criteria, 278 full-text articles were assessed for eligibility. Among these, 16 studies were non-randomized controlled trials, 55 did not include a control group, 23 did not use LBL in control group, 11 used other teaching methods in addition to LBL in the control group, 16 used other teaching methods in addition to the PBL or PBL + CBL in the intervention group, 34 did not compare the baseline indicators between the two groups, 4 had duplicate data, and 44 did not provide the required data. One article included two groups based on graduate and non-graduate students, so counted into two studies. Thus, a total of 75 articles (76 studies) were included in this meta-analysis [19–93] (Additional file 1: Table A1).

### Study characteristics

The characteristics of these 76 included studies, published in Chinese between 2010 and 2019, are listed in Table 1. The sample size of these studies was 10–108 residents in the intervention group and 12–107 in the control group, and the pooled sample size was 4597 (intervention group = 2323, control group = 2274). The included studies covered 26 specialties (23 Internal Medicine, 8 Neurology, 3 Emergency Medicine, 4 Pediatrics, 16 Surgery, 4 Anesthesiology, 3 Obstetrics and Gynecology, 3 Ophthalmology, 2 Dentistry, 4 Medical Sonography, 1 Psychiatry, 2 Radiation Oncology, 2 Radiology, and 1 Traditional Chinese Medicine). All the studies described the baseline information about residents and tutors in both groups. For residents, 68 studies were matched for age, 67 were matched for sex, 44 were matched for educational background (EB), 29 were matched for department entrance exam scores (DS), 3 were matched for clinical working hours, 1 was matched for PBL experience, 1 was matched for the duration of intervention, and 1 was matched for the year of residency training. For tutors, 30 studies were matched for professional titles (PT), 14 were matched for teaching experience (TE), 4 were matched for educational background (EB), 9 described that the tutors were the same in both groups, 5 described that tutors in both groups received PBL teaching training, and 6 described that tutors in both groups performed collective lesson preparation (CLP). In 47 studies, PBL was performed in the intervention groups, and in 29 studies, PBL + CBL was conducted in the intervention groups. The residents were in the first or second year of residency training, as mentioned in 7 studies, while 18 studies described the duration of intervention, 3 weeks–8 months.

**Table 1:** Characteristics of the included studies

Study ID	Year	Study type	Sample size(I/C)	Specialty of residents	Residents matched for	Tutors matched for	Interventions	Which year of residency training	Duration of intervention	Outcome measures
Li M et al. (2019) [19]	2019	RCT	46(23/23)	Internal Medicine	Age, Sex, EB, DS	The same tutors	PBL	Not described	Not described	KS
Wang N et al. (2017) [20]	2017	RCT	74(37/37)	Internal Medicine	Age, Sex	Not described	PBL	Not described	Not described	TS
Gao WQ et al. (2019) [21]	2019	RCT	50(25/25)	Cardiology	Age, Sex, EB (Bachelor Degree)	TE	PBL	Not described	Two months	KS, PS, QS
Liu J et al. (2017) [22]	2017	RCT	56(28/28)	Gastroenterology	Age, Sex, DS	PT, TE, Recieved PBL teaching training, CLP	PBL	Not described	Three months	TS, QS
Gulina Abra and Wang XM (2019) [23]	2019	RCT	70(35/35)	Gastroenterology	Age, Sex, DS	Not described	PBL	Not described	Three months	KS, SS, PS, QS
Wei DM et al. (2017) [24]	2017	RCT	133(68/65)	Gastroenterology	Age, Sex	PT, TE	PBL	Not described	Not described	KS, PS, QS
Hou H et al. (2019) [25]	2019	RCT	84(42/42)	Gastroenterology	Age, Sex	Not described	PBL	Not described	Not described	KS, PS, QS
Chang BC et al. (2018) [26]	2018	RCT	48(24/24)	Nephrology	Sex, EB	Not described	PBL	Not described	Not described	KS
Tang JL et al. (2019) [27]	2019	RCT	40(20/20)	Haematology	Age, Sex, DS	The same tutor	PBL	Not described	Not described	TS
Wang YY et al. (2017) [28]	2017	RCT	42(21/21)	Infectious Disease	Age, Sex, EB	PT, TE	PBL	Not described	Not described	KS, SS, PS, TS, QS
Xie SS et al. (2018) [29]	2018	RCT	215(108/107)	Infectious Disease	Age, Sex, EB (Graduate students)	Not described	PBL	Not described	Not described	TS, QS
Zhao D (2019) [30]	2019	RCT	54(29/25)	Critical Care Medicine	Age, Sex	Not described	PBL	Not described	Not described	KS, SS, PS
Xing JY et al. (2017) [31]	2017	RCT	84(42/42)	Critical Care Medicine	Age, Sex, EB	PT, TE	PBL	Not described	Two months	KS, PS
Liu ZG et al. (2012) [32]	2012	RCT	35(18/17)	Neurology	DS	PT, CLP	PBL	Not described	Not described	TS, QS
Wang Y (2015) [33]	2015	RCT	32(17/15)	Neurology	Age, Sex, DS	Not described	PBL	Not described	Three months	KS, PS, TS
Huang YX (2019) [34]	2019	RCT	48(24/24)	Neurology	Age, Sex, EB, DS	PT	PBL	Not described	Three months	TS, QS
Shi JQ and Jiang T (2018) [35]	2018	RCT	65(33/32)	Neurology	Age, Sex, EB	PT, Recieved PBL teaching training	PBL	Not described	Six months	KS, SS, PS
Cheng J et al. (2015)a [36]	2015	RCT	28(16/12)	Neurology	DS, EB (Graduate students)	PT, CLP	PBL	Not described	Not described	TS, QS
Cheng J et al. (2015)b [36]	2015	RCT	24(10/14)	Neurology	DS, EB (Bachelor Degree)	PT, CLP	PBL	Not described	Not described	TS, QS
Huang JX et al. (2016) [37]	2016	RCT	60(30/30)	Emergency Medicine	Age, Sex, EB, DS	PT, EB, TE	PBL	Not described	Not described	KS, SS, QS
Lin F et al. (2016) [38]	2016	RCT	82(43/39)	Pediatrics	Age, Sex, EB	PT	PBL	Not described	Eight months	KS, SS, PS
Jiang CQ et al. (2018) [39]	2018	RCT	71(35/36)	General Surgery	Age, Sex, EB (Graduate students), No PBL experience	PT, TE, Recieved PBL teaching training	PBL	Not described	Four months	KS, SS, PS
Huang XX (2018) [40]	2018	RCT	50(25/25)	Gastrointestinal Surgery	Age, Sex	PT, TE	PBL	Not described	Two months	KS, SS, PS
Ge ST et al. (2018) [41]	2018	RCT	80(41/39)	Gastrointestinal Surgery	Age, Sex, EB	The same tutors, PT, TE, Recieved PBL teaching training	PBL	First-year residents	Two months	KS, SS, PS
Guan YB et al. (2018) [42]	2018	RCT	60(30/30)	Urology	Age, Sex, EB	Not described	PBL	Not described	Not described	TS, QS
Zhang JL et al. (2019) [43]	2019	RCT	40(20/20)	Urology	Age, Sex, EB	Not described	PBL	Not described	Not described	KS, SS
Ma Y et al. (2018) [44]	2018	RCT	80(40/40)	Orthopedics	Age, Sex	Not described	PBL	Not described	Not described	KS
Zhou P et al. (2014) [45]	2014	RCT	60(30/30)	Neurosurgery	Age, Sex, EB, DS	PT	PBL	Not described	Not described	TS
Lin Y and Jiang H (2014) [46]	2014	RCT	26(12/14)	Anesthesiology	Age, Sex, DS	The same tutors	PBL	Not described	Not described	KS, SS
Li ZR et al. (2019) [47]	2019	RCT	32(16/16)	Anesthesiology	Age, Sex	Not described	PBL	Not described	Not described	KS, SS
Jiang J et al. (2017) [48]	2017	RCT	48(24/24)	Anesthesiology	Age, Sex, EB, DS	Not described	PBL	Not described	Not described	KS, SS
Xin WQ et al. (2017) [49]	2017	RCT	64(32/32)	Anesthesiology	Age, Sex, DS	The same tutors	PBL	Not described	Not described	TS
Zheng LJ and Guo LS (2018) [50]	2018	RCT	34(18/16)	Obstetrics and Gynecology	Age, Sex, DS	PT, EB, TE	PBL	Not described	Not described	KS, SS, QS
Han J and Yan XL (2017) [51]	2017	RCT	40(20/20)	Ophthalmology	Age, Sex, DS	Not described	PBL	Not described	Not described	TS
Chen JL (2018) [52]	2018	RCT	24(12/12)	Ophthalmology	Age, Sex	Not described	PBL	Not described	Not described	KS, PS
Liu GX et al. (2018) [53]	2018	RCT	83(55/28)	Orthodontics	Age, Sex	Not described	PBL	Not described	Not described	KS, QS
Chen HB et al. (2019) [54]	2019	RCT	46(23/23)	Medical Sonography	Age, Sex, EB	Not described	PBL	First-year or second-year residents	Three months	KS, SS, QS
Yang JC et al. (2015) [55]	2015	RCT	50(25/25)	Medical Sonography	Age, Sex, EB, Years of residency training	Not described	PBL	First-year or second-year residents	Not described	KS, SS
Fan X et al. (2016) [56]	2016	RCT	80(40/40)	Medical Sonography	Age, Sex, EB (Graduate students), DS	The same tutors, PT	PBL	Not described	Not described	TS, QS
Dong FL and Fan QM (2015) [57]	2015	RCT	28(14/14)	Medical Sonography	Age, Sex, EB	TE	PBL	Not described	Not described	KS, PS
Lu Y et al. (2014) [58]	2014	RCT	143(75/68)	Internal Medicine	DS	PT, EB, TE, Recieved PBL teaching training	PBL	Not described	Not described	QS
Wang Y et al.(2018) [59]	2018	RCT	80(40/40)	Infectious Disease	Age, Sex, EB	PT	PBL	Not described	Not described	QS
Yi XL et al. (2017) [60]	2017	RCT	100(50/50)	Pediatrics	Age, Sex, DS	PT, TE	PBL	Not described	Not described	QS
Zhang J et al. (2017) [61]	2017	RCT	92(46/46)	Thoracic Surgery	Age, Sex, EB (Graduate students), DS	CLP	PBL	First-year residents	One months	QS
Chen JX et al. (2015) [62]	2015	RCT	120(60/60)	Urology	Age, Sex, EB (Bachelor Degree)	CLP	PBL	First-year residents	Not described	QS
Ma Y and Zhang X (2019) [63]	2019	RCT	60(30/30)	Ophthalmology	Age, Sex	Not described	PBL	Not described	Not described	QS
Wang Z et al. (2019) [64]	2019	RCT	94(47/47)	Radiology	Age, Sex, EB	Not described	PBL	Not described	Not described	QS
Yang XY and Jia F (2019) [65]	2019	RCT	91(46/45)	Cardiology	Age, Sex, EB	Not described	PBL + CBL	Not described	Not described	PS, QS
Jiang H et al. (2017) [66]	2017	RCT	62(31/31)	Cardiology	Age, Sex	Not described	PBL + CBL	Not described	Not described	TS
Lin FN et al. (2017) [67]	2017	RCT	82(40/42)	Cardiology	Age, Sex	Not described	PBL + CBL	First-year residents	Not described	TS, QS
Shi XJ et al. (2018) [68]	2018	RCT	40(20/20)	Gastroenterology	Age, Sex, Duration of intervention	Not described	PBL + CBL	First-year residents	Not described	KS, PS, QS
Jin L et al. (2018) [69]	2018	RCT	48(24/24)	Gastroenterology	Age, Sex, EB, DS	PT	PBL + CBL	Not described	Four months	TS, QS
Hu XL et al. (2017) [70]	2017	RCT	40(20/20)	Endocrinology	Age, Sex, DS	PT	PBL + CBL	Not described	Not described	TS
Wu Y et al. (2018) [71]	2018	RCT	92(47/45)	Critical Care Medicine	EB	PT	PBL + CBL	Not described	Two months	KS, PS
Li SX et al. (2019) [72]	2019	RCT	60(30/30)	Emergency Medicine	Age, Sex	The same tutors	PBL + CBL	Not described	Not described	KS, PS, QS
Liu FS et al. (2018) [73]	2018	RCT	48(24/24)	Emergency Medicine	Age, Sex, EB	Not described	PBL + CBL	Not described	Not described	KS, SS, PS
Song YH (2019) [74]	2019	RCT	40(20/20)	Psychiatry	Age, Sex	Not described	PBL + CBL	Not described	Not described	KS, PS
Guan XL et al. (2018) [75]	2018	RCT	46(23/23)	Neurology	Age, Sex, EB	The same tutors	PBL + CBL	Not described	Not described	TS
Liu HH and Xiao GD (2018) [76]	2018	RCT	28(14/14)	Neurology	Age, Sex, DS	Not described	PBL + CBL	Not described	Not described	KS, PS
Wan QQ (2018) [77]	2018	RCT	66(33/33)	Pediatrics	Sex, EB, Clinical working hours	Not described	PBL + CBL	Not described	Not described	TS
Zhao Y et al. (2017) [78]	2017	RCT	43(20/23)	Pediatrics	Age, Sex, EB, Clinical working hours	PT, TE	PBL + CBL	Not described	Not described	KS, SS, PS
Li H et al. (2019) [79]	2019	RCT	80(40/40)	General Surgery	Age, Sex, EB	Not described	PBL + CBL	Not described	Not described	KS, QS
Shi GY (2019) [80]	2019	RCT	80(40/40)	General Surgery	Age, Sex, EB	Not described	PBL + CBL	Not described	Not described	TS
Liu F and He JG (2018) [81]	2018	RCT	52(26/26)	General Surgery, Gastroenterology	Age, DS	PT	PBL + CBL	Not described	Four to five months	KS, SS, PS
Hu GD (2019) [82]	2019	RCT	40(20/20)	Cardiothoracic Surgery	Age, Sex, EB	Not described	PBL + CBL	Not described	Not described	KS, SS, PS
Zhang L et al. (2017) [83]	2017	RCT	32(16/16)	Cardiothoracic Surgery	Age, EB, DS	Not described	PBL + CBL	Not described	Not described	KS, SS, PS, QS
Xu L et al. (2017) [84]	2017	RCT	40(20/20)	Neurosurgery	Age, Sex, EB	Not described	PBL + CBL	Not described	Not described	KS, SS
Zhao L et al. (2019) [85]	2019	RCT	48(24/24)	Obstetrics and Gynecology	Age, Sex, EB	Not described	PBL + CBL	Not described	Not described	TS
Ji H (2018) [86]	2018	RCT	40(20/20)	Obstetrics and Gynecology	Age, DS	PT	PBL + CBL	Not described	Not described	TS
Jiang HJ et al. (2019) [87]	2019	RCT	62(31/31)	Radiology	Age, Sex, DS	PT	PBL + CBL	Not described	Not described	KS, PS, QS
Wang HY and Kong LL (2019) [88]	2019	RCT	46(23/23)	Radiation Oncology	Age, Sex, EB (Graduate students)	PT	PBL + CBL	Not described	Not described	TS, QS
Xiao L et al. (2018) [89]	2018	RCT	40(20/20)	Radiation Oncology	Age, Sex, EB (Graduate students), DS, Clinical working hours	The same tutors, PT	PBL + CBL	Not described	Not described	TS, QS
Chen Z et al. (2019) [90]	2019	RCT	50(25/25)	Traditional Chinese Medicine	Age, Sex, EB, DS	PT, EB	PBL + CBL	Not described	One month	TS
Wang Y and Hao W (2017) [91]	2017	RCT	50(25/25)	Cardiology	Age, Sex, EB	Not described	PBL + CBL	Not described	Three weeks	QS
Wang BQ et al. (2016) [92]	2016	RCT	48(24/24)	General Surgery	Age, Sex	Not described	PBL + CBL	Not described	Not described	QS
Xu P and Li CJ (2016) [93]	2016	RCT	48(24/24)	General Dentistry	EB	Not described	PBL + CBL	Not described	Not described	QS

*KS* knowledge scores, *SS* skill scores, *TS* total scores, *PS* practical skills, *QS* questionnaire surveys *DS* department entrance exam scores, *EB* educational background, *TE* teaching experience, *CLP* collective lesson preparation, *PT* professional titles, *I* intervention group, *C* control group

There were 40, 22, 27 and 27 studies in KS, SS, PS and TS respectively. QS were used as the outcomes to evaluate several abilities among the theoretical knowledge mastery, clinical diagnostic thinking, teamwork ability, ability to analyze and solve problems, communication ability, learning interest, self-directed learning ability, hands-on ability, ability to consult documents, learning efficiency, and satisfaction with teaching in 37 studies.

### Study quality

**Fig. 2:**
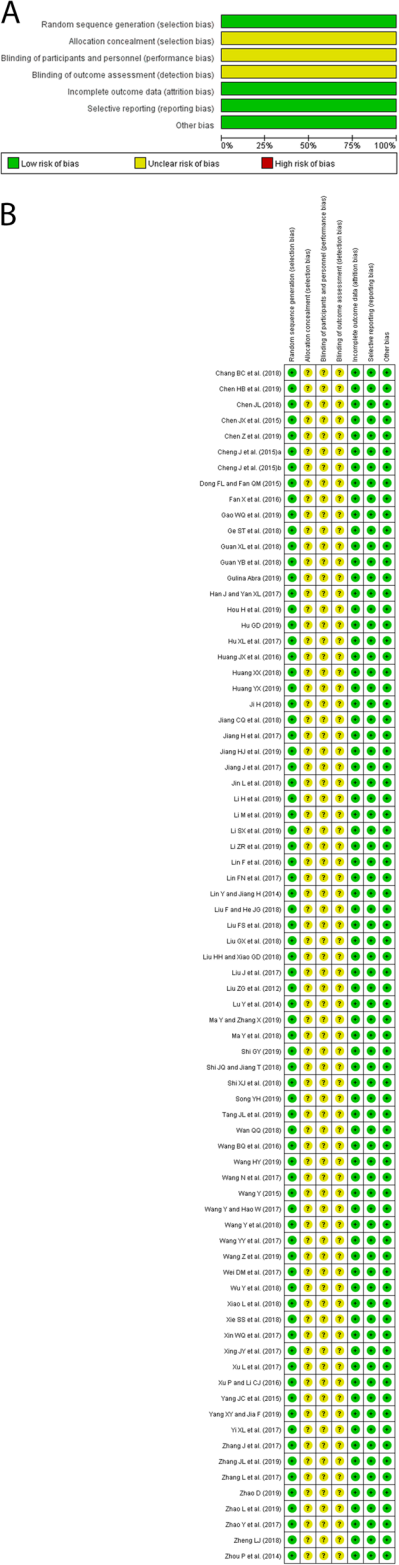
Risk of bias assessment. **A** Risk of bias graph as percentages for all included studies; (**B**) Risk of bias summary for each included study

All the included studies were assessed for the risk of bias (Fig. 2). The studies were designed as randomized controlled trials, and the results were reported adequately. All studies were free of selective reporting and other biases. The allocation concealment and blinding were not stated in these studies.

### Effects of interventions (PBL and PBL + CBL) on KS

**Fig. 3:**
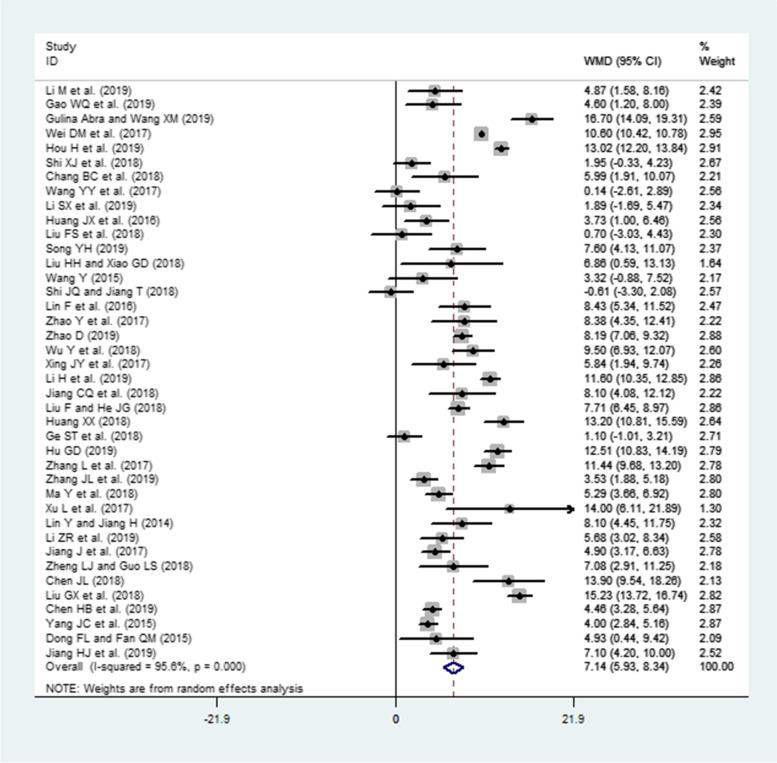
Forest plot for the effects of interventions (PBL and PBL + CBL) on knowledge scores compared to the LBL group

A total of 40 publications involving 2190 residents (intervention group = 1111 and LBL group = 1079) reported KS. Because a high heterogeneity was observed across these studies (I^2^ = 95.6%, *P* < 0.0001), the random-effects model was used. The pooled effect size showed a significant difference in KS (WMD = 7.14, 95% CI: 5.93–8.34, *P* < 0.0001) in favor of the intervention group compared to the LBL group (Fig. 3).

### Effects of interventions (PBL and PBL + CBL) on SS

**Fig. 4:**
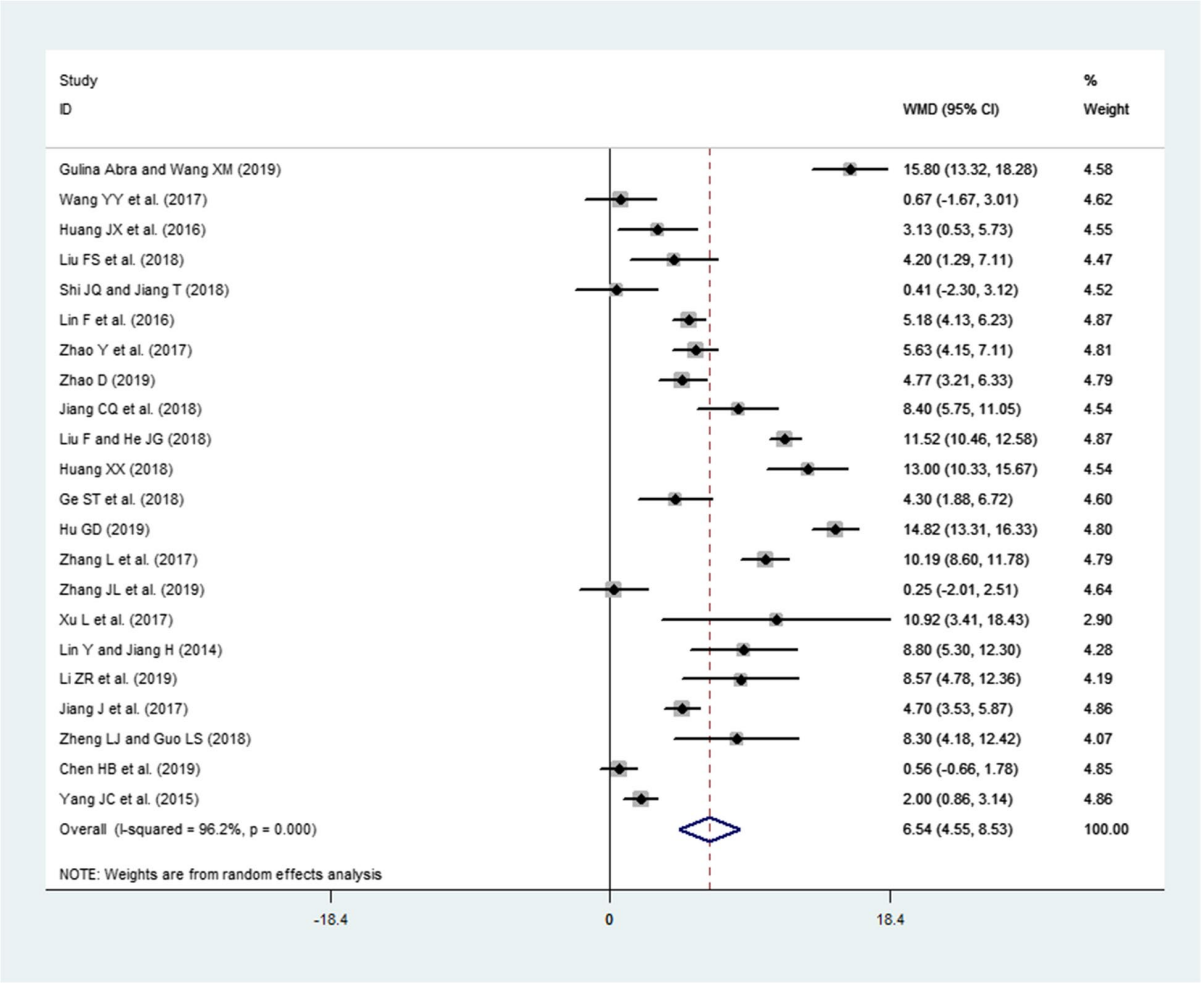
Forest plot for the effects of interventions (PBL and PBL + CBL) on skill scores compared to the LBL group

A total of 22 publications involving 1096 residents (intervention group = 547, LBL group = 549) reported SS. Because a high heterogeneity was observed across these studies (I^2^ = 96.2%, *P* < 0.0001), the random-effects model was used. The pooled effect size showed a significant difference in skill scores (WMD = 6.54, 95% CI: 4.55–8.53, *P* < 0.0001) in favor of the intervention group compared to the LBL group (Fig. 4).

### Effects of interventions (PBL and PBL + CBL) on PS assessments

**Fig. 5:**
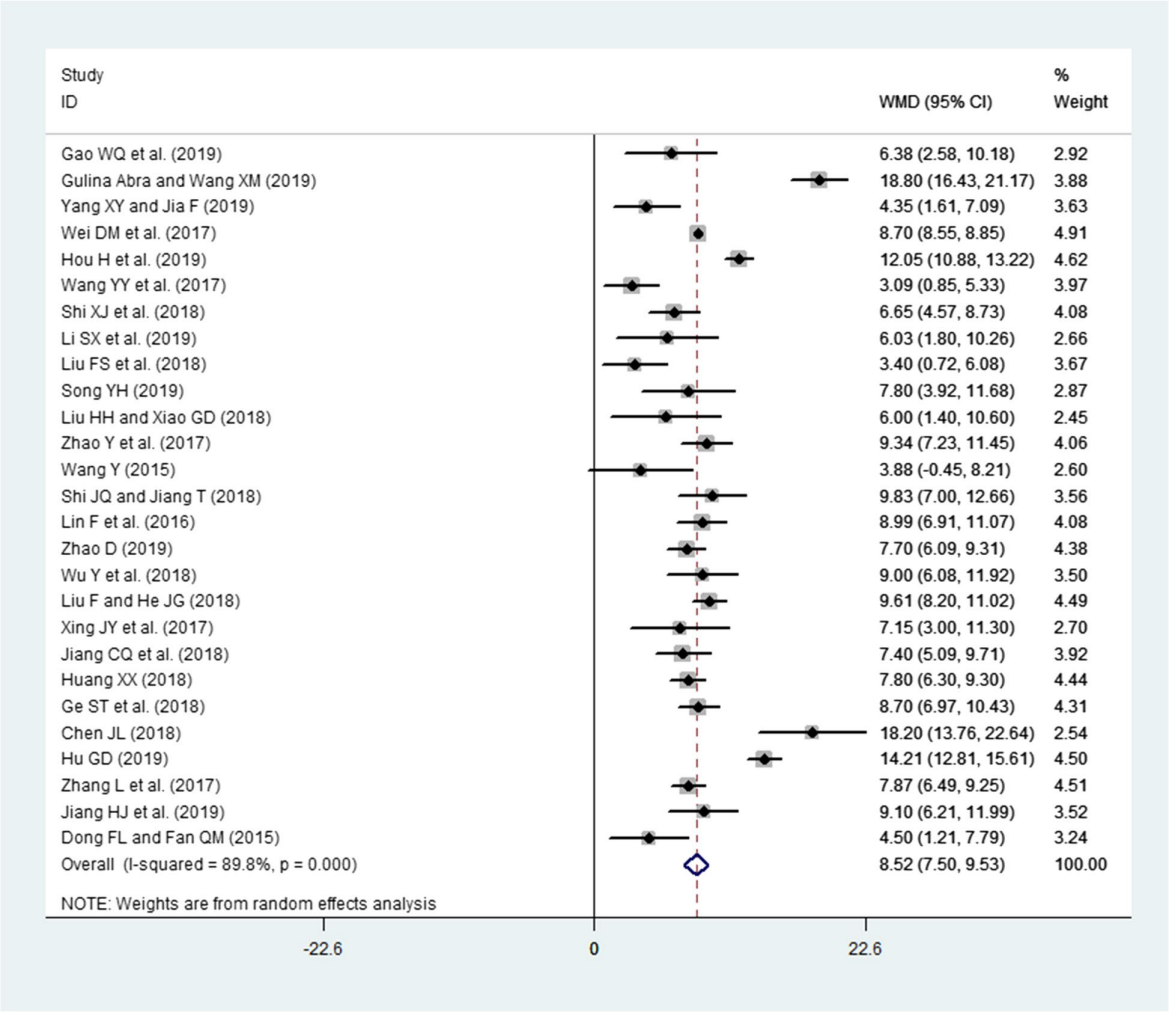
Forest plot for the effects of interventions (PBL and PBL + CBL) on practical skills assessments compared to the LBL group

A total of 27 publications involving 1568 residents (intervention group = 787, LBL group = 781) reported PS assessments. Because a high heterogeneity was observed across all these studies (I^2^ = 89.8%, *P* < 0.00001), the random-effects model was used. The pooled effect size showed a significant difference in PS assessments (WMD = 8.52, 95% CI: 7.50–9.53, *P* < 0.0001) in favor of the intervention group compared to the LBL group (Fig. 5).

### Effects of interventions (PBL and PBL + CBL) on TS

**Fig. 6:**
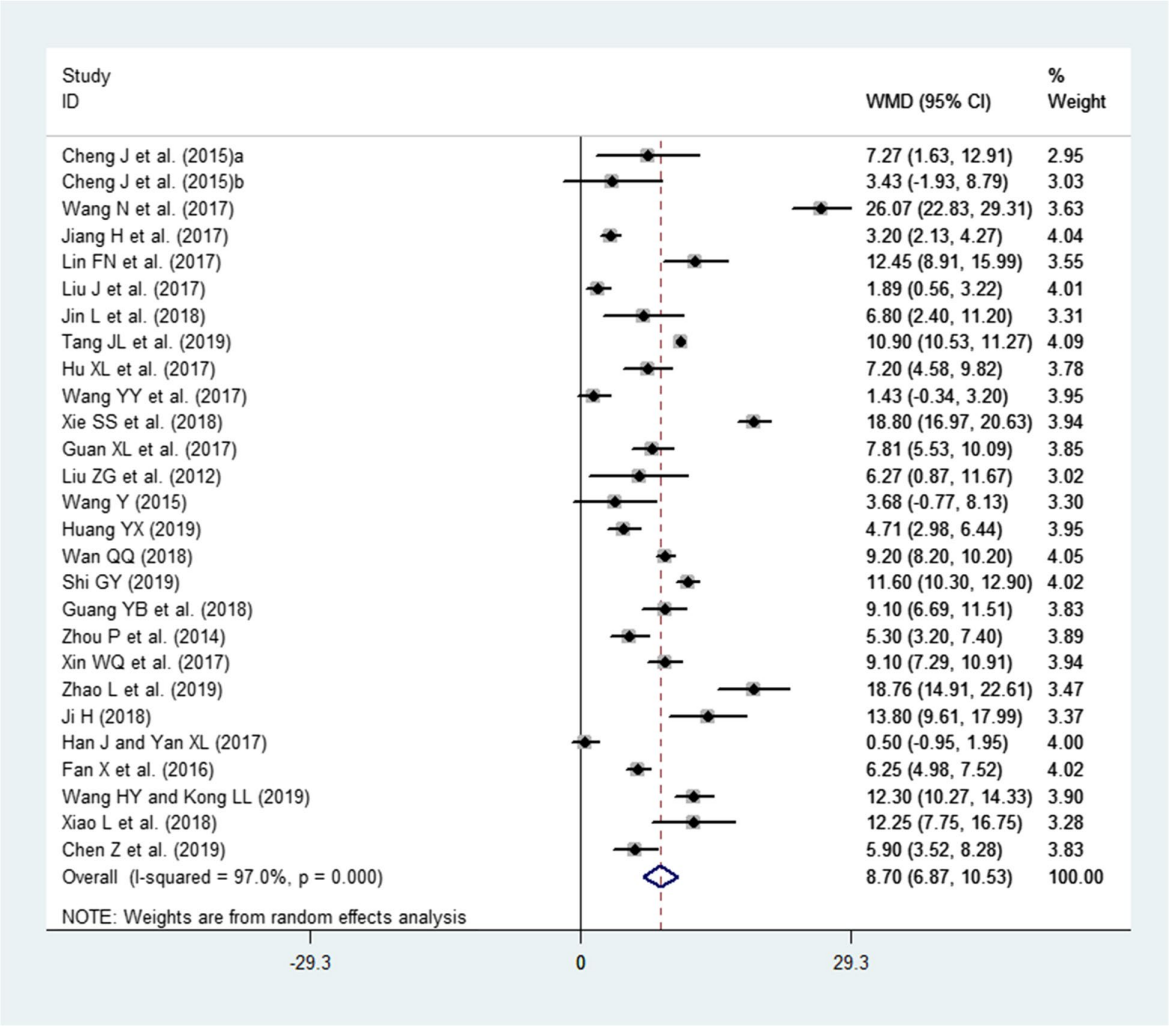
Forest plot for the effects of interventions (PBL and PBL + CBL) on total scores compared to the LBL group

A total of 27 publications involving 1542 residents (intervention group = 770, LBL group = 772) reported TS. Because a high heterogeneity was observed across these studies (I^2^ = 97%, *P* < 0.00001), the random-effects model was used. The pooled effect size showed a significant difference in the total score (WMD = 8.70, 95% CI: 6.87–10.53, *P* < 0.0001) in favor of the intervention group compared to the LBL group (Fig. 6).

### Subgroup analysis of outcome measurements

In order to explore the sources of heterogeneity, this study conducted a subgroup analysis of teaching methods and departments. The teaching methods were divided into PBL group *vs*. LBL group and PBL + CBL group *vs*. LBL group. The teaching departments were divided into non-surgery, surgery, and diagnostic. The data are shown in Table 2. Only diagnostic imaging did not display a statistical significance in SS, while the data from the other subgroups differed significantly compared to the LBL group. However, the heterogeneity was not reduced significantly.

**Table 2:** Subgroup analysis of outcome measurements

Factors		Studies (n)	Sample size (I)	Sample size (C)	WMD	95% CI
Knowledge Scores						
Teaching methods	PBL	27	783	750	6.86	(5.31–8.41)
	PBL + CBL	14	328	329	7.74	(5.64–9.84)
Departments	Non-surgery	20	598	594	6.23	(4.53–7.93)
	Surgery	16	420	392	8.80	(6.63–10.97)
	Diagnostic imaging	4	93	93	4.55	(3.58–5.53)
Skill Scores						
Teaching methods	PBL	16	421	420	5.41	(3.55–7.26)
	PBL + CBL	6	126	129	9.53	(6.37–12.69)
Departments	Non-surgery	8	226	229	4.99	(2.46–7.52)
	Surgery	12	273	272	8.59	(5.97–11.20)
	Diagnostic imaging	2	48	48	1.30	(-0.11–2.71)
Practical Skills Assessments						
Teaching methods	PBL	15	473	467	8.90	(7.46–10.33)
	PBL + CBL	12	314	314	7.94	(6.04–9.84)
Departments	Non-surgery	18	567	562	7.95	(6.59–9.31)
	Surgery	7	175	174	10.19	(7.93–12.45)
	Diagnostic imaging	2	45	45	6.87	(2.36–11.38)
Total Scores						
Teaching methods	PBL	15	447	447	7.67	(4.79–10.56)
	PBL + CBL	12	323	325	9.92	(7.57–12.27)
Departments	Non-surgery	16	466	468	8.24	(5.66–10.83)
	Surgery	7	196	196	9.55	(5.39–13.72)
	Diagnostic imaging	3	83	83	10.07	(5.28–14.86)

*PBL* Problem-based learning

*CBL* Case-based learning

*I* Intervention group

*C* Control group

### Effects of interventions assessed by QS

The questionnaire (Table 3.) showed that the intervention group is superior to the LBL group with respect to theoretical knowledge mastery, clinical diagnostic thinking, teamwork ability, ability to analyze and solve problems, ability to consult documents, learning interest, satisfaction with teaching, and learning efficiency, with a statistically significant difference. On the other hand, the differences in improving self-directed learning ability, communication ability, and hands-on ability were not statistically significant.

**Table 3:** Effects of interventions assessed by questionnaires

Research indicators	Studies (n)	Sample size (I)	Sample size (C)	ORs	95% CI
Theoretical knowledge mastery	13	543	535	1.26	(1.05–1.52)
Clinical diagnostic thinking	11	502	491	1.42	(1.17–1.72)
Teamwork ability	14	546	537	2.35	(1.65–3.34)
Learning interest	21	775	765	1.49	(1.27–1.74)
Self-directed learning ability	14	562	552	1.32	(0.99–1.77)
Ability to analyze and solve problems	15	489	479	1.60	(1.31–1.95)
Ability to consult documents	2	58	58	1.90	(1.04–3.49)
Satisfaction with teaching	25	859	824	1.34	(1.16–1.55)
Communication ability	3	117	109	1.49	(0.97–2.28)
Hands-on ability	6	157	156	1.39	(0.98–1.96)
Learning efficiency	4	289	281	1.46	(1.13–1.88)

### Sensitivity analysis

Owing to high heterogeneity, sensitivity analysis was implemented to evaluate the reliability of the results. After excluding the study with the largest weight [24], the pooled effect size was in favor of the intervention group (WMD 7.03, 95% CI: 5.58–8.50, *P* < 0.00001) for KS and did not change the effects observed in the primary analysis. Conversely, after excluding the study with the largest weight [24, 27, 81], the pooled effect size in SS, PS assessment, and TS was in favor of the intervention group (WMD 6.28, 95% CI: 4.33–8.22, *P* < 0.00001; WMD 8.45, 95% CI: 7.08–9.82, *P* < 0.00001; WMD 8.61, 95% CI: 6.56–10.67, *P* < 0.00001). No single study was found to significantly influence the overall pooled WMD, indicating the stability of our results.

### Publication bias

**Fig. 7:**
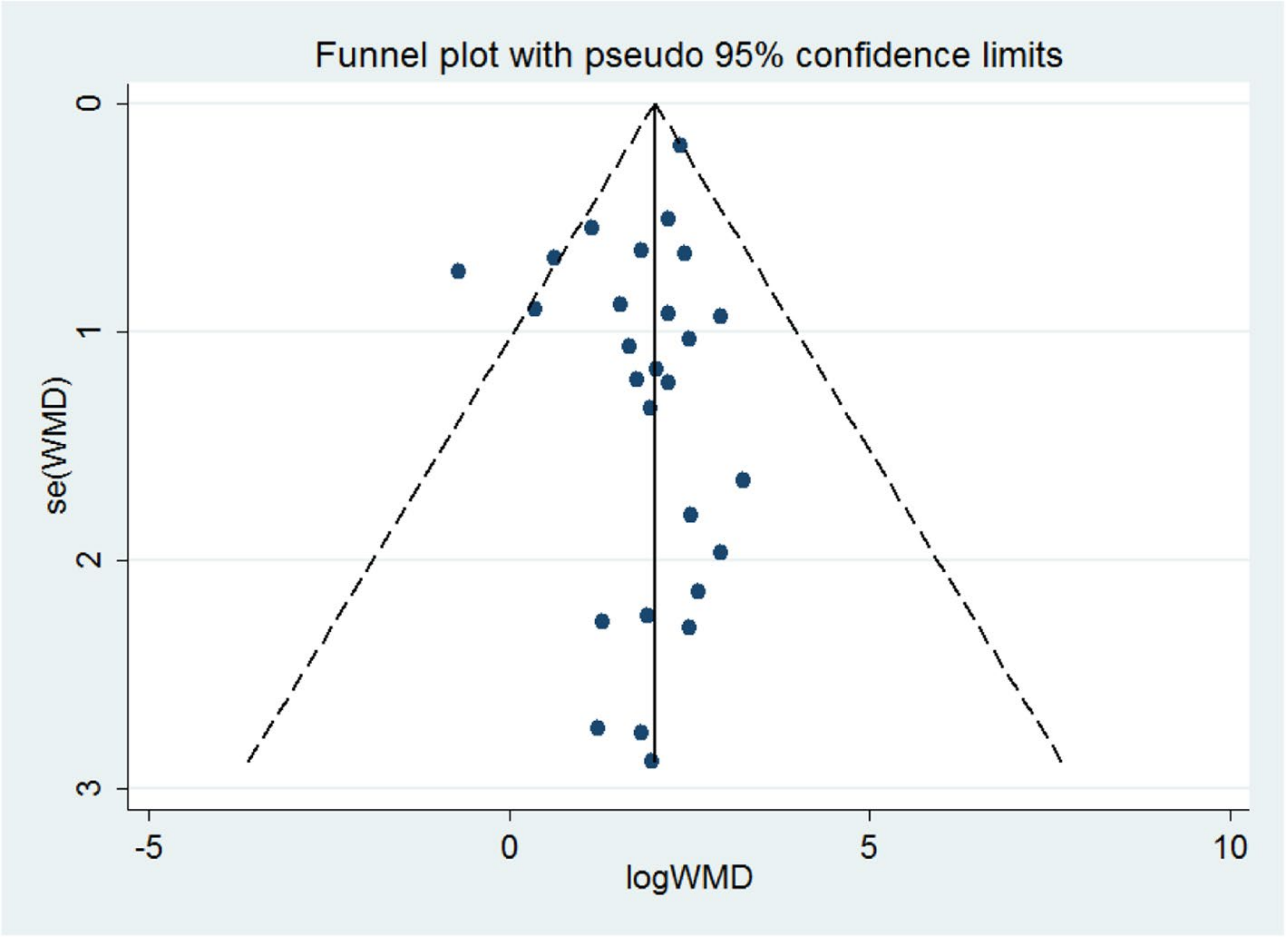
Funnel plot analysis for total scores

The evaluation of publication bias was conducted using a funnel plot for the 27 studies with respect to the total scores (Fig. 7). The shape of the funnel plot did not show asymmetry, indicating the absence of any publication bias. Also, no significant bias was detected using the Begg’s rank correlation test (Z = 0.21, *P* = 0.835) and Egger’s linear regression test (t = -1.23, *P* = 0.228).

## Discussion

In the current meta-analysis, the results showed that the residents in the PBL-based teaching groups have better scores in knowledge, skill, PS assessments, and TS than those in LBL groups, indicating that PBL could help residents to better master the medical theory knowledge, operational skills, analyze and diagnose cases and overall capacity than LBL. The QS showed that PBL-based strategies are superior to LBL in improving residents’ theoretical knowledge mastery, clinical diagnostic thinking, teamwork ability, ability to analyze and solve problems, ability to consult documents, learning interest, and learning efficiency. Also, the residents exhibited more satisfaction with teaching for PBL-based strategies than for LBL. However, PBL-based strategies did not significantly improve self-directed learning ability, communication ability, and hands-on ability.

### Clinical cases are used as problems of PBL in medical education

PBL and CBL are student-centered, focusing on students’ subjective initiative, cultivating their ability to find and solve problems. However, they are different teaching strategies since PBL commonly presents complex, open-ended problems about topics previously unknown to the students and develops problem-solving skills through self-teaching and discussion, even solutions may vary from group to group. However, CBL takes clinical cases as the starting point, uses relevant knowledge and theory to analyze these cases, solves the clinical problems efficiently, and improves the clinical ability. Over the years, PBL in medical education has shifted towards a case-focused approach, wherein the only problems considered by the students are patient cases, and the discussion phase is reduced to a minimalistic list of questions or learning objectives, and reporting diagnoses and medical facts is emphasized. This method prompted the medical educators to wonder about the purpose of PBL and switch to the CBL method. Other educators using the term CBL referred to a case as the problem [94, 95]. Thus, all the studies in this meta-analysis encompassed clinical cases as trigger problems. To avoid incomplete inclusion of the literature due to differences in understanding of the concepts and to accurately assess the role of PBL teaching methods in SRT, we included studies involving PBL or PBL combined with CBL. Also, heterogeneity analysis was carried out according to the teaching method, but the results did not reduce heterogeneity (see Sect. 4.3 for details).

### Analysis of the role of PBL in the cultivation of residents’ abilities

Thomas et al. [96] found that residents who attended a PBL medical school (PBL group) performed significantly better on standardized tests than on those who attended a traditional medical school in obstetrics and gynecology residency program, which is consistent with our finding of knowledge acquisition. Sun et al. [97] found that compared to the traditional teaching model, problem- and simulator-based learning for lumbar puncture training can develop overall surgical skills in neurology residents, which is consistent with the current finding about operational skills. In subgroup analysis, we found that PBL based teaching shows no advantage on operational skills in diagnostic imaging. In another study, Yue et al. [98] found that the integration of PBL and LBL teaching modes in the education of imaging diagnosis education produced a good teaching effect, which needs to be substantiated with additional studies. The residents’ ability to analyze and diagnose cases include history taking, physical examination, and analysis of inspection results is crucial for residency training. According to our findings in PS assessments, PBL-based teaching enabled the development of this critical capability. The above analysis of different capabilities showed overall positive capacity according to TS.

The residents showed a preference for PBL-based strategies. The positive effect of PBL on mastery of theoretical knowledge, clinical diagnostic thinking, teamwork ability obtained by our analysis which is in agreement with previous studies may explain this result well [99, 100]. The survey of self-directed learning showed some improvement in residents, but the improvement is not significant in the PBL group. The reason for this could possibly be associated with residents dealing with various problems of patients every day, has developed stronger self-directed learing ability in clinical practice than undergraduates. Zhang et al. [61] discussed that the PBL group requires time and effort for preparation before the class, while the existing teaching facilities could not fully meet their needs. In addition, the students in China received “spoon-feeding” education for a long time, and the literature retrieval level was limited. Therefore, improving the level of teaching hardware, and giving guidance on the retrieval methods is needed. The PBL teaching mode could not improve communication ability, and the analysis by Sanghee et al. [101] might explain the related factors, because of the cultural climate of Asian countries, students were reluctant to express their opinion to a tutor who has authority and felt uncomfortable to challenge classmates’ views. It’s worth noting that only three included studies assessed the communication ability as the intervention outcome measures. This may imply that insufficient attention was paid to this ability when conducting PBL in Chinese SRT. Therefore, the guidance and encouragement of the tutor is necessary for the development of effective communication, not only between the resident and the tutor, but also among the training residents.

### Analysis of heterogeneity

An obvious heterogeneity was detected among the included studies for KS, SS, PS assessments, and TS. In order to explore the source of heterogeneity, the teaching methods and departments were analyzed in subgroups, but the results did not show reduced heterogeneity. The reasons for the analysis of high heterogeneity are as follows.

First, the comprehensive ability of the resident teacher is an important contributor to the training of qualified residents, as well as a factor related to the quality of SRT. The difference in the teaching level exerts an influence on the teaching effect. Although all the studies in this meta-analysis are carried out in hospitals affiliated to medical schools or equivalent providing high-level medical and health services, most studies do not mention the situation of the teachers. In addition, teachers should strengthen the study of new theories and methods. Although teachers are familiar with PBL teaching mode with an extensive attempt of PBL in undergraduate teaching, only a few described that teachers had received the PBL teaching training. The existing studies also lack the supervision and evaluation of teachers. The difference in the teaching level of teachers may be one of the causes of heterogeneity.

Second, the residents' basic quality and learning experience are different [102]. Some are SRT trainees applying for Master of Medicine degree during the 3 years simultaneously, which might have a strong learning aspiration and ability. Only a few studies mentioned previous clinical working hours and whether residents had been exposed to PBL teaching methods. Therefore, we deduced that the learning ability, clinical work experience, and PBL training experience of residents are the potential causes of heterogeneity.

Third, the present evaluation method formulated by the training department could not form a unified evaluation system for each specialty according to its characteristics. In addition, for capacity assessment, a long-term evaluation should be more suitable. Carrero et al. [103] found that the effectiveness of lecture and case/problem-based learning differed only slightly in terms of improving immediate clinical capacity in the first year Anesthesiology residents while suggesting that there should be an appropriate tool to determine the effect of different teaching methodologies on the long-term retention of knowledge, skills, attitudes and clinical competence. Therefore, lack of a unified evaluation system may be one cause for heterogeneity.

### Limitations

In addition to the factors mentioned above that may cause heterogeneity, the quality of the articles is also one of the limitations of this meta-analysis. Although all the studies included in this meta-analysis were randomized controlled trials, none of them described the allocation concealment in detail, and no blinding method was used which was caused by the objective limitations in teaching. The small sample size in some of the studies was also one of the limitations. Besides, at present, there is no unified scale to assess the above mentioned skills of residents in the included studies. For example, QS in each study were designed by the training department itself based on the contents of previous reported questionnaires and the goal of SRT. So the inconsistencies of the assessment among these included studies were also one of the limitations. Another limitation about QS is that because the results are assessed in a yes or no form, resulting in missing data and inefficient data use. Additionally, there were many possible factors influencing the effect of PBL, stratified analysis should be conducted to test the heterogeneity.

## Conclusions

The present meta-analysis shows that the PBL teaching method is more effective than LBL in the mastery of medical theory knowledge, operational skills, analysis and diagnosis of cases, and the overall capacity of SRT in mainland China. However, it shows no advantage on operational skills in diagnostic imaging. QS displayed the positive effects of PBL on theoretical knowledge mastery, clinical diagnostic thinking, teamwork ability, ability to analyze and solve problems, ability to consult documents, learning interest, and learning efficiency. The QS results also indicated that residents showed more satisfaction with teaching for PBL-based strategies than for LBL. However, PBL-based strategies had not improved significantly with respect to self-directed learning ability, communication ability, and hands-on ability. This meta-analysis provided a systematic and comprehensive analysis and achieved the training contents suitable for the PBL teaching model. To the aspects which the results did not show any improvement, the effect of other teaching methods should be discussed in the future. According to our results, the basic data could be obtained for a detailed design and performance of suitable teaching methods for various specialties and abilities in Chinese SRT. Nonetheless, the heterogeneity of the included studies needs to be considered, and further well-designed studies are needed to confirm our findings.

## Supplementary Information

**Additional file 1: Table A1. **The references of included studies.

## Data Availability

The datasets used and/or analysed during the current study available from the corresponding author on reasonable request.

## Abbreviations

SRT: Standardized residency training
PBL: Problem-based learning
LBL: Lecture-based learning
CBL: Case-based learning
QS: Questionnaire surveys
KS: Knowledge scores
SS: Skill scores
PS: Practical skills
TS: Total scores
EB: Educational background
DS: Department entrance exam scores
PT: Professional titles
TE: Teaching experience
CLP: Collective lesson preparation
