# Supplementary material for: Problem-based or lecture-based learning, old topic in the new field: a meta-analysis on the effects of PBL teaching method in Chinese standardized residency training
Source: BMC Med Educ. 2022 Mar 31;22:221. doi: 10.1186/s12909-022-03254-5 (PMC8974027; doi:10.1186/s12909-022-03254-5)
Supplement: Supplementary file 1 — Additional file 1: Table A1. The references of included studies. [file 12909_2022_3254_MOESM1_ESM.docx]

Table A1 The references of included studies

| Study ID | References (In Chinese) |
| --- | --- |
| Li M et al. (2019) | Li M, Liang B, Li YW, Yang QY, Gao F. (2019). Application of PBL teaching method in the teaching rounds of general practice residency training. China Higher Medical Education, 4, 100-101. |
| Wang N et al. (2017) | Wang N, Yan W, Yan T. (2017).Application of PBL teaching method in internal medicine base for standardized residency training. Research analysis, 13, 324. |
| Gao WQ et al. (2019) | Gao WQ, Wang LB, Yang GY, Zhao XH, Li YH. (2019).Application of PBL teaching mode in cardiovascular skill training of general residents. Medical Information, 32(7), 20-21. |
| Liu J et al. (2017) | Liu J, Ling Y, Wang J, Xi CH, Yuan C. (2017). Application of PBL teaching method in resident standardized training in the Department of Gastroenterology. China Modern Doctor, 55(33), 141-144. |
| Gulina Abra and Wang XM (2019) | Gulina Abra and Wang XM. (2019). Discussion on the application value of PBL teaching method in standardized residency training in gastroenterology. Healthful Friend,10, 154. |
| Wei DM et al. (2017) | Wei DM, He GN, Yang WJ. (2017). Effect of applying PBL teaching method in standardized residency training in gastroenterology. Chinese Journal of Rural Medicine and Pharmacy, 24(3), 67-68. |
| Hou H et al. (2019) | Hou H, Liu HM, Liu J. (2019). Analysis of the application effect of applying PBL teaching in the standardized residency training in gastroenterology. China Health Care & Nutrition, 13, 394. |
| Chang BC et al. (2018) | Chang BC, Chen WD, Zhang Y, Wu XP, Guo YL. (2018). Evaluate the teaching effect of PBL by application Mini-CEX in resident standardization training of chief physician. Journal of Qiqihar Medical University, 39(5), 583-585. |
| Tang JL et al. (2019) | Tang JL, Shi M, Huang CL, Li XM, Zhang L. (2019).Application of WeChat and PBL teaching in general practice training. Continuing Medical Education, 33(10), 17-19. |
| Wang YY et al. (2017) | Wang YY, Zhou QY, Zhu LY, Zhong LH, Lu BL, Cheng Y, Yao H, Fan J, Yu L. (2017). The influence of teaching model of WeChat combined with the PBL to the teaching quality of standardized training of residents. China Continuing Medical Education, 9(15), 10-12. |
| Xie SS et al. (2018) | Xie SS, Zhang Y, Zuo WZ. (2018).The application of PBL teaching method in the standardized residency training in the Infectious Disease Department. J Mod Med Health, 34(7), 1103-1105. |
| Zhao D (2019) | Zhao D. (2019). Research on the application of PBL teaching model based on diagnosis and treatment guidelines to the standardized residency training in Intensive Care Medicine. Health Education, 123-124. |
| Xing JY et al. (2017) | Xing JY, Han XN, Yuan ZY, Sun XX, Xu XF, Sun YB. (2017).Application of clinical cases related problem-based-learning in critical care medical education. Chin J Med Edu Res, 16(6), 614-618. |
| Liu ZG et al. (2012) | Liu ZG, Gan J, Wang XJ. (2012). Practice of PBL teaching method in rotation teaching of the Department of Neurology. China Higher Medical Education, 11,20-21,77. |
| Wang Y (2015) | Wang Y. (2015).Application of PBL combined with LBLm teaching model in standardized residency training of neurology. Anhui Medical Journal, 36(7), 891-893. |
| Huang YX (2019) | Huang YX. (2019).Application of PBL teaching method in standardized training of neurological residents. China Continuing Medical Education, 11(31), 67-69. |
| Shi JQ and Jiang T (2018) | Shi JQ, Jiang T. (2018). The application of problem-based learning education model in the standardized training for neurology residents and its effect evaluation. Journal of Jiangsu Institute of Commerce, 6,59-61. |
| Cheng J et al. (2015)a | Cheng J, Chen W, Gan J, Liu ZG. (2015). Practice of PBL teaching method in the teaching of resident neurology in different educational backgrounds. China Higher Medical Education, 2, 94-95. |
| Cheng J et al. (2015)b | Cheng J, Chen W, Gan J, Liu ZG. (2015). Practice of PBL teaching method in the teaching of resident neurology in different educational backgrounds. China Higher Medical Education, 2, 94-95. |
| Huang JX et al. (2016) | Huang JX, Zhu HM, Wu LQ, Zhang YN. (2016). Application of PBL teaching mode in standardized residency training of emergency. Journal of Traditional Chinese Medicine Management, 24(23), 84-85. |
| Lin F et al. (2016) | Lin F, Yu QY, Zheng JH, Pan XX, Hang JG. (2016). Application of PBL teaching mode on standardized training of pediatric residents. Hospital Management Forum, 33(10), 53-55. |
| Jiang CQ et al. (2018) | Jiang CQ, Zhu PS, Shi Y, Xiang WJ, Ge ST, Zhang ZB, Zuo LG. (2018). Effect analysis of using PBL teaching mode in resident standardization training for professional postgraduate students majored in general surgery. Journal of Qiqihar Medical University, 39(21), 2554-2557. |
| Huang XX (2018) | Huang XX. (2018). The application of PBL teaching model in standardized training for resident in gastrointestinal surgery. Journal of Anhui Health Vocational & Technical College, 17(6), 109-110,113. |
| Ge ST et al. (2018) | Ge ST, Shao RR, Zhang ZB, Liu ML, Jiang CQ. (2018). Application of problem-based learning educational model in resident standardization training of gastrointestinal surgery. China Continuing Medical Education, 10(7), 1-3. |
| Guan YB et al. (2018) | Guan YB, Nong SJ, Cai B, Ma LM, Xing QW, Geng JS. (2018). A practical study on PBL teaching method in standardized training of urology residents.Continuing Medical Education. 2018, 32(12):38-40. |
| Zhang JL et al. (2019) | Zhang JL, Lu QZ, Zhang ZY, Hou JQ. (2019). The analysis of efficacy of PBL teaching method in standardization training of residents in urology. Education Teaching Forum, 50, 209-210. |
| Ma Y et al. (2018) | Ma Y, Liu ZW, Zhu X, Ma JY, Sui JT, Li Y. (2018). Application of PDCA cycle combined with PBL teaching method in training of orthopedics residents. Journal of Bingtuan Medicine, 3, 64-66. |
| Zhou P et al. (2014) | Zhou P, Wang XQ, Li XY, Li ST. (2014). Application of PBL teaching model in standardized training of neurosurgery clinical base. Education Teaching Forum, 33, 107-108. |
| Lin Y and Jiang H (2014) | Lin Y, Jiang H. (2014). Application of problem-based learning as a teaching model in difficult airway management training using a simulator. Chinese Journal of Medicinal Guide, 16(6), 1103-1104. |
| Li ZR et al. (2019) | Li ZR, Geng XB, Sun ML, Ma HN, Liu M, Zhou CY. (2019). Comparison of the effects of PBL and LBL in standardized residency training of anesthesiology. Our Health, 12，123. |
| Jiang J et al. (2017) | Jiang J, Lin Y, Zheng YC, Huang Y, Jiang H. (2017). Problem-based learning versus lecture-based learning in standardized training of residents in anesthesiology. Chinese Medical Record, 18(10), 98-101. |
| Xin WQ et al. (2017) | Xin WQ, Yan Z, Shi CN, Guo SL, Li AX, Zhang YX. (2017). Effect of WeChat platform combined with problem-based learning teaching method when applied to the standardized training for residents in a Department of Anesthesiology. Chin J Anesthesiol, 37(4), 392-395. |
| Zheng LJ and Guo LS (2018) | Zheng LJ, Guo LS. (2018). Application of PBL teaching methods in the standardized resident training of obstetrics and gynecology. China Continuing Medical Education, 10(18), 5-7. |
| Han J and Yan XL (2017) | Han J, Yan XL. (2017). Comparisons of teaching methods in standardized training for ophthalmic residents. Int Eye Sci, 17(5), 904-907. |
| Chen JL (2018) | Chen JL. (2018). The application value of three teaching modes of PBL, TBL and LBL in the standardized training of ophthalmology residents. Contemporary Medical Symposium, 16(19), 192-194. |
| Liu GX et al. (2018) | Liu GX, Zhong AX, Pan XB, Peng LH, Lu QF, Pan MZ, Li RT. (2018). The application and thinking of PBL teaching model in the standardization training of resident in clinical prosthodontics. Journal of Minimally Invasive Medicine, 13(5), 665-667. |
| Chen HB et al. (2019) | Chen HB, Ma Q, Liu ST. (2019). Application of PBL teaching method in standardized training of ultrasonic medical residents. China Continuing Medical Education, 11(2), 1-2. |
| Yang JC et al. (2015) | Yang JC, Ma J, Wang P, Wang L, Lu JS, Du L. (2015). Application of PBL teaching method in the clinical teaching of resident doctors in Ultrasound Medicine Department. Chinese Medical Record, 16(9), 79-81. |
| Fan X et al. (2016) | Fan X, Song T, Hou XQ, Dong J, Yang LF. (2016). Application of PBL teaching mode in standardized residency training in ultrasonic base. Health Vocational Education, 34(10), 73-74. |
| Dong FL and Fan QM (2015) | Dong FL, Fan QM. (2015). The application of PBL teaching mode in the standardization training of residents in ultrasound medicine. Fork Lore, 123-124. |
| Lu Y et al. (2014) | Lu Y, Yu DH, Zhang B, Zhang HZ, Wang MH. (2014). Application of problem-based learning model in general practical standard training. GP Chinese General Practice, 17(16), 1880-1883. |
| Wang Y et al.(2018) | Wang Y, Deng BC, Liu P. (2018). The application of PBL in resident standardization training in the Department of Infectious Diseases. China Continuing Medical Education, 10(14), 9-11. |
| Yi XL et al. (2017) | Yi XL, Li XL, Jiang Y. (2017). Application of PBL teaching mode in standardized training of pediatric residents. Journal of Qiqihar University of Medicine, 38(16), 1936-1937. |
| Zhang J et al. (2017) | Zhang J, Yang KP, Hou XS, Ge XQ, Qiao MH, Hou L. (2017). The applying of problem-based learning in standardized training of residents of thoracic surgery. Chin J Med Edu, 37(5), 779-782,796. |
| Chen JX et al. (2015) | Chen JX, An HY, Wang F. (2015). Research of PBL teaching method in urology surgical practice. Chinese Medicine Modern Distance Education of China, 13(4), 111-112. |
| Ma Y and Zhang X (2019) | Ma Y, Zhang X. (2019). Application effect of PBL teaching in standardized training of ophthalmology residents. Jilin Medical Journal, 40(3), 679-680. |
| Wang Z et al. (2019) | Wang Z, Su DK, Lai SL, Jin GQ, Xie D, Kang W, Zhao Y, Yang J. (2019). The application of PBL teaching method in the standardized training of residents in oncology medical imaging. Guangxi Medical Journal, 41(12), 1597-1599. |
| Yang XY and Jia F (2019) | Yang XY, Jia F. (2019). The application of CBL combined with PBL method in the ward round teaching of residents in Department of Cardiology. China Continuing Medical Education, 11(36), 24-26. |
| Jiang H et al. (2017) | Jiang H, Najina Wugeti, Wang K, Wang LP, Ailiman Mahemuti. (2017). Application of PBL and CBL dual-track teaching in standardized training of residents in cardiology. Xinjiang Medical Journal, 47(6), 686-687. |
| Lin FN et al. (2017) | Lin FN, Huang LH, Xiong SQ, Zheng F, Zhan P, Li CY, Lin W, Qiu CY. (2017). Application of PBL and CBL combined teaching method in the diagnosis and treatment of coronary heart disease. Chinese Journal of Geriatric Care, 15(3), 126-127. |
| Shi XJ et al. (2018) | Shi XJ, Liang XP, Yi He lasi，He FP, Fan XT. (2018). Evaluation of the effect of CBL combined with PBL teaching model in standardized training of resident doctors. Medical Information, 31(6), 16-18. |
| Jin L et al. (2018) | Jin L, Fang L, Li SY, Zhu WC, Dai FL, Dun SS. (2018). The Application of PBL and CBL combined with holistic integrated medicine in the training of digestive endoscopy. China Continuing Medical Education, 10(30), 3-6. |
| Hu XL et al. (2017) | Hu XL, Chen WD, Sun WH, Yu L, Shi ZM, Jin GX. (2017). Application of the teaching method combining PBL with CBL in resident standardization training in the Department of Endocrinology. Chinese Journal of General Practice, 15(7), 1236-1238. |
| Wu Y et al. (2018) | Wu Y, Zhang M, Wang XC, Zhao YJ. (2018). The application of problem/case-based learning teaching method in standardized training of residents in the Department of Critical Care Medicine. China Continuing Medical Education, 10(29), 16-19. |
| Li SX et al. (2019) | Li SX, Yin LL, Wang M, Wang LL, Zhao S, Zhao JL. (2019). The use of PBL combined with CBL in emergency resident standardized training. China Continuing Medical Education, 11(34), 28-30. |
| Liu FS et al. (2018) | Liu FS, Wang SM, Liu J, Zheng XC, Li SF, Zhu Y, Shen W, Pan HH, Fang XL. (2018). The application effect of CBL combined with PBL teaching method oriented by post competence in clinical teaching of traditional Chinese medicine emergency. China Higher Medical Education, 6, 81-82. |
| Song YH (2019) | Song YH. (2019). Application of two teaching methods in the training of psychiatric residents. China Continuing Medical Education, 11(33), 48-50. |
| Guan XL et al. (2018) | Guan XL, Zheng Y, Bi S, Lu XL, Yang H, Tao Y, Hu J. (2018). Application of PBL combined with CBL teaching method in the multiple sclerosis teaching for training of residents. Studies of Trace Elements and Health, 35(3), 71-73. |
| Liu HH and Xiao GD (2018) | Liu HH, Xiao GD. (2018). Application of CBL combined with PBL teaching model in clinical standardized training of neurology residents. Chinese Journal of Coal Industry Medicine, 21(5), 553-556. |
| Wan QQ (2018) | Wan QQ, Yan PL, Wu F. (2018). Discussion on teaching methods of tracheal intubation in pediatric resident standardized training. China Modern Doctor, 56(28), 132-134. |
| Zhao Y et al. (2017) | Zhao Y, Zhang CX, Guo YY. (2017). Application of PBL combined with medical record in the standardized training of pediatric resident physicians. Medicine and Health Education, 60-61. |
| Li H et al. (2019) | Li H, Li SL, Li YM, Liu QF, Liu Y. (2019). Analysis of the effect of CBL, PBL combined with simulation training method in clinical teaching of general surgery. China Continuing Medical Education, 11(18), 16-18. |
| Shi GY (2019) | Shi GY. (2019). Application of PBL combined with CBL teaching method in standardized training of general surgery residents. Health Must-Read Magazine, 35, 261. |
| Liu F and He JG (2018) | Liu F, He JG. (2018).Application combined PBL with CBL in the standardized training of resident physicians in the Digestive Diseases Department.Journal of Nongken Medicine, 40(1), 76-79. |
| Hu GD (2019) | Hu GD. (2019). Application of CBL and PBL combined with simulated teaching method in the standardized training of residents in Cardiothoracic Surgery Department. China Health Care & Nutrition, 29(27), 316-317. |
| Zhang L et al. (2017) | Zhang L, Tang Z, Liang B, Li XJ, Gong HY, Wang B. (2017). Application of CBL and PBL combined with simulated training method in the teaching of clinical resident physician in Cardiothoracic Surgery department. Chinese Journal of General Practice, 15(7), 1233-1235. |
| Xu L et al. (2017) | Xu L, Xiao SW, You CY, Tang HB, Zhang XJ, Wu HT, Zhao HX, Dai Y, Chen XZ, Xie MX. (2017). Research and analysis on the teaching methods of standardized training for surgical residents in neurosurgery rotation. Medicine and Health Education, 92-93,96. |
| Zhao L et al. (2019) | Zhao L, Du R, Ma R, Ma CL. (2019). The effectiveness of applying PBL combined with CBL teaching in the standardized training of residents in obstetrics and gynecology. International Infectious Disease (Electronic Edition), 8(3), 224-225. |
| Ji H (2018) | Ji H. (2018). The application of PBL and CBL double track teaching in standardized training of obstetrics and gynecology resident. China Continuing Medical Education, 10(7), 3-5. |
| Jiang HJ et al. (2019) | Jiang HJ, Feng GL, Jia YL, Jiang H, Zhang MY, Xu HL. (2019). Application of PBL combined with CBL teaching model in standardized training of residents in Radiology Department. China Higher Medical Education, 2, 108-109. |
| Wang HY and Kong LL (2019) | Wang HY, Kong LL. (2019). Application of CBL and PBL teaching methods in the teaching of residents receiving standardized training in the Tumor Radiotherapy Department. The Science Education Arrical Collects, 465, 100-102. |
| Xiao L et al. (2018) | Xiao L, Mayinuer Aili, Bao YX. (2018). Application of problem-based learning combined with case based learning in standardized training of residents in Department of Radiation Oncology. Chinese Medical Record, 19(8), 76-78. |
| Chen Z et al. (2019) | Chen Z, Xu L, Jin Y, Kou ZJ, Wang XD. (2019). A study on the application of PBL combined with CBL in standardized training for residents of Tuina Department. Journal of New Chinese Medicine, 51(7), 318-320. |
| Wang Y and Hao W (2017) | Wang Y, Hao W. (2017). Effect evaluation of CBL combined with PBL teaching mode in the clinical teaching of Cardiovascular Department. Chinese Medical Record, 18(9), 83-85. |
| Wang BQ et al. (2016) | Wang BQ, Xue F, Zhang GQ, Ding W. (2016). Application of teaching method based on questions and cases in the training of residents in oncology surgery. China Higher Education, 5, 67-68. |
| Xu P and Li CJ (2016) | Xu P, Li CJ. (2016). Application of PBL and CBL teaching in the standardized training of oral resident. China Continuing Medical Education, 8(33), 3-4. |
